# The Multiplication of Metropolitan Hospitals

**Published:** 1892-07-23

**Authors:** 


### The Multiplication of Metropolitan Hospitals.

From a Correspondent.

It is often alleged, somewhat too hastily, against the hospital system (or, as some would say, want of system) in the metropolis, that there is a constant and increasing number of these institutions opened year by year in London, and that this increase is by no means necessary or desirable. I should be the first to deprecate the production of mushroom-like growths of this kind, but it is as well for the public to know exactly what the alleged increase amounts to, and I therefore gladly publish the subjoined table, prepared by Mr. Howgrave Graham, Secretary of the Hospital for Epilepsy and Paralysis, Regent's Park, showing from the information contained in "Burdett's Hospital Annual," 1891-92, and in The Hospital, not only the number of hospitals which have come into existence during the last ten and during the last twenty years, but also the date of establishment, the number of beds, and the expenditure of each institution, as far as such particulars have been furnished, by the hospitals themselves, to the Editor of the "Annual."

It appears from the following tables that in the last 10 years 18, and in the last 20 years 32 new "hospitals" have been originated, but an examination into the nature of the institutions, as indicated by the particulars appended to their names, will show that many of them are exceptional in character, or are purely local, and would scarcely justify a sweeping complaint of a recent large increase in the number of "hospitals." For instance, the existence of an Italian hospital is as justifiable as that of a French or German hospital. The Memorial Cottage Hospital, attached to, and, it is presumed, dependent upon, a particular Mission, can scarcely be said to compete with other hospitals, while the same may be said of the Mildmay Mission Hospital, Her Majesty's Infirmary for Sick Children (in connection with Dr. Barnardo's Homes), and St. Raphael's (Roman Catholic) Hospital for Men, while the Cheyne Hospital for Sick and Incurable Children, the Home and Infirmary for Sick Children, and the Sydenham Hospital and Home for Incurable Children evidently aim at objects not within the scope of other hospitals for children. The need for some of the remaining institutions in the list is less apparent, and, without in any way wishing to detract from the (no doubt) excellent work done by these, it is a little difficult, in face of the existence for more than twenty years of other hospitals for the same purpose, to see the *raison d'être*, much less the need for starting, such institutions as the London Skin Hospital, the London Throat Hospital, the St. Andrew's Eye and Ear Hospital, the Central London Throat and Ear Hospital, the West End Hospital for Diseases of the Nervous System, the London Massage and Galvanic Hospital (which, in its advertisement in your columns, invites the patients at the West End Hospital to transfer their patronage to the newer institution), the Queen's Jubilee Hospital, the Gordon Hospital for Fistula, and the British Hospital for Mental Disorders ("Forbes Winslow Memorial")—though, with respect to the last named, there may be a hitherto unsuspected advantage in providing for mental cases not sufficiently bad for seclusion in proper asylums treatment other than that afforded by the skilled and numerous staff of the existing hospitals for nervous diseases.

These lists may be classified into skin, throat and ear, and nervous system, and it contains the names of seven hospitals for diseases of these classes, while at the time of the foundation of these new institutions there were already in existence three hospitals of each class. The existence of other new institutions may be justifiable by a certain amount of "specialism," but specialism may easily degenerate into "faddism." There is always, however, much to be said in favour of locality. Towards the central and west of London, there is such a congestion of medical charities that the establishment of new institutions in other parts of the vast metropolis is, on this ground at least, excusable; but on the other hand many of the new hospitals have only added to the local congestion referred to.

My object in making these remarks on the table is rather to lead your readers to form their own conclusions than to press upon them any views of mine, but the subject is one which has so often elicited criticism that it is well for the public to know the facts, as far as I have been able to supply them, otherwise I might refer in much stronger terms to some of the hospitals enumerated in the table.

As far as can be ascertained from the tables, the 32 hospitals make up 800 beds at an annual expenditure of over £47,000 or £58 per bed per annum.

Hospitals Established Since 1881.

| Name and Address of Hospital. | Date of Establishment | No. of Beds Available. | Expenditure. |
|---|---|---|---|
| London Skin Hospital, 47, Cranbourne St. | 1887 | None | £471 |
| Queen's Jubilee Hospital, Richmond Road, South Kensington | 1887 | 20 | § |
| London Throat Hospital for Diseases of the Throat, Nose, and Ear, 204, Gt. Portland Street | 1887 | 4 | § |
| St. Gabriel's Hospital for Infants, 117, Grosvenor Road | 1885 | 12 | ¶ |
| Italian Hospital, 41, Queen Square, Bloomsbury | 1884 | 20 | 914 |
| Memorial Cottage Hospital, Mildmay Pk., N. | 1883 | 31 | |
| Paddington Green Hospital for Sick Children, Paddington Green, W. | 1883 | 27 | 2,594 |
| Gordon Hospital for Fistula and other Diseases of the Rectum, 278, Vauxhall Bridge Road, S.W. | 1884 | 11 | 1,418 |
| Woolwich and Plumstead Cottage Hospital, Shooter's Hill | 1889 | 12 | 562 |
| Miller Hospital, Greenwich | 1882 | 23 | 3,402 |
| St. Andrew's Eye and Ear Hospital, 67, Wells Street, W. | 1882 | * | * |
| Norwood Cottage Hospital, Norwood, S.E. | 1882 | 16 | 916 |
| Clapham Maternity Hospital, 74, Jeffreys Road, S.W. | 1889 | 14 | 454 |
| British Hospital for Mental Disorders, "Forbes Winslow Memorial," 208, Euston Road | 1890 | | |
| Lady Gomm Memorial Mission House and Accident Hospital, Hawkestone Road, Rotherhithe | 1883 | 6 | 718 |
| London Massage and Galvanic Hospital, 55, Weymouth Street | 1891 or '2 | † | |
| St. John's Hospital, 1, 2, and 3, Montague Place, Poplar | 1884 | ‡ | 1,043 |
| West Ham, Stratford, and South Essex Hospital and Dispensary, Stratford | 1890 | 34 | 2,000 |

Hospitals Established Between 1871 and 1881.

| Name and Address of Hospital. | Date of Establishment | No. of Beds Available. | Expenditure. |
|---|---|---|---|
| | | | £ |
| Chelsea Hospital for Women, Fulham Road, S.W. | 1871 | 60 | 4,050 |
| North-West London Hospital, 18, 20, and 22, Kentish Town Road, N.W. | 1878 | 47 | 2,971 |
| West-end Hospital for Diseases of the Nervous System, Paralysis, and Epilepsy, 73, Welbeck Street, W. | 1878 | 50‖ | 2,747 |
| Mildmay Missions Hospital and Dispensary, Turville Street, Bethnal Green | 1877 | 32 | 2,644 |
| Central London Throat and Ear Hospital, Gray's Inn Road | 1873 | 14, 3‖ | 1,979 |
| Her Majesty's Hospital for Waif Children (Dr. Barnardo's), 13, Stepney Causeway, E. | 1876 | 74 | ** |
| St. Raphael's (Roman Catholics) Hospital for Men, 3, Sydney St., Fulham Rd., S.W | 1876 | 15 | ** |
| Cheyne Hospital for Sick and Incurable Children, Cheyne Walk, Chelsea, S.W. | 1874 | 24 | 4,977 |
| London Temperance Hospital, Hampstead Road, N.W. | 1873 | 76 | 4,376 |
| St. Saviour's Cancer Hospital (for Women only as In-patients—Paralysis, Cancer, Nervous Affections, Diseases of Women, Osnaburgh Street, N.W. | 1872 | 36 | 3,618 |
| New Hospital for Women, 222, Marylebone Road | 1872 | 42 | 2,204 |
| Home and Infirmary for Sick Children, Lower Sydenham | 1872 | 45 | 1,452 |
| Blackheath and Charlton Cottage Hospital, Blackheath | 1880 | 20 | 832 |
| Hospital and Home for Incurable Children, 2, Maida Vale | 1875 | 30 | 1,414 |

* No report published, maintained on provident principles. † No particulars in the Annual. ‡ No return. § Not stated. ¶ Not published. ‖ Cots. ** Not stated.

